# Synchronous Presentation of Chronic Myelomonocytic Leukemia and Multiple Myeloma in a Treatment‐Naïve Patient

**DOI:** 10.1155/crh/5531249

**Published:** 2026-06-12

**Authors:** Moutaz W. Sweileh, Naim Qamhia, Amal Batta, Hisham Asaad, Razan Yousef Odeh, Mamoun Swaileh

**Affiliations:** ^1^ Faculty of Medicine and Allied Medical Sciences, An-Najah National University, Nablus, State of Palestine, najah.edu; ^2^ Department of Pathology, An-Najah National University Hospital, Nablus, State of Palestine, najah.edu; ^3^ Department of Pathology & Laboratory Medicine, Royal Liverpool University Hospital, Liverpool, UK, nhs.uk; ^4^ Department of Internal Medicine, An-Najah National University Hospital, Nablus, State of Palestine, najah.edu

**Keywords:** chronic myelomonocytic leukemia, hematopathology, multiple myeloma, synchronous hematologic neoplasms

## Abstract

**Background:**

Chronic myelomonocytic leukemia (CMML) is a clonal myelodysplastic/myeloproliferative neoplasm characterized by persistent peripheral blood monocytosis and dysplastic bone marrow morphology. Multiple myeloma (MM) is a plasma cell malignancy defined by clonal plasma cell proliferation and myeloma‐defining events. The synchronous occurrence of CMML and MM is exceptionally rare, particularly in treatment‐naïve and relatively young patients.

**Case Presentation:**

We present a 42‐year‐old‐male presenting with progressive fatigue and leukocytosis. Laboratory evaluation revealed marked absolute monocytosis (16.4 × 10^9^/L), anemia, and neutropenia. Bone marrow examination demonstrated an expanded abnormal monocytic population, further confirmed by multiparametric flow cytometry. Conventional cytogenetic analysis revealed monosomy 7, supporting the presence of a clonal myeloid neoplasm consistent with CMML. Concurrently, bone marrow biopsy showed 10%–15% plasma cells. Serum protein electrophoresis identified an IgG kappa monoclonal paraprotein (3.5 g/dL), and imaging showed a lytic bone lesion and biopsy‐provided extramedullary plasmacytoma, meeting the diagnostic criteria of symptomatic MM. The patient has no prior exposure to cytotoxic therapy. He received one cycle of azacitidine with subsequent worsening cytopenias, underscoring therapeutic complexity.

**Conclusions:**

This case describes a rare concurrent presentation of CMML and MM in a young, treatment‐naïve patient. The combination of persistent monocytosis, dysplastic marrow findings, and monosomy 7 supported the presence of clonal myeloid process coexisting with plasma cell myeloma. Recognizing concurrent hematologic malignancies is essential, as diagnostic overlap and treatment interactions present substantial management challenges. Additional molecular characterization of similar cases may elucidate potential pathogenetic connections and guide tailored therapeutic approaches.

## 1. Background

According to the WHO, chronic myelomonocytic leukemia (CMML) is a clonal hematopoietic stem cell neoplasm that falls under the myelodysplastic/myeloproliferative overlap syndromes. It is characterized by persistent absolute (≥ 0.5 × 10^9^/L) and relative (≥ 10%) peripheral blood monocytosis accompanied by dysplastic and/or proliferative bone marrow features [1]. The median age at diagnosis is roughly 73–75 years, with a slight male predominance. Cytogenetic abnormalities are identified in about 20%–30% of cases and commonly include trisomy 8, loss of the Y chromosome, monosomy 7 or del (7q), trisomy 21, and complex karyotypes. CMML demonstrates considerable biologic and clinical heterogeneity and is frequently associated with recurrent cytogenetic and molecular abnormalities [2].

Plasma cell myeloma, also known as multiple myeloma (MM), is a clonal plasma cell neoplasm characterized by clonal proliferation of plasma cells. The diagnosis requires ≥ 10% clonal bone marrow plasma cells or biopsy‐proven bony or extramedullary plasmacytoma in association with one or more myeloma‐defining events. These include evidence of end‐organ damage attributable to the plasma cell disorder (hypercalcemia, renal failure, anemia, or lytic bone lesions; CRAB criteria) or specific biomarkers of malignancy such as ≥ 60% clonal bone marrow plasma cells, serum involved/uninvolved free light chain (FLC) ratio ≥ 100 (provided involved FLC is ≥ 100 mg/L and urine monoclonal protein is ≥ 200 mg/24 h), or > 1 focal lesion on magnetic resonance imaging. The median age at diagnosis is 66–70 years [3, 4].

The coexistence of CMML and MM is exceedingly rare. A review of the literature identified approximately 10 reported cases of CMML coexisting with MM or a related plasma cell dyscrasia; among these, the majority represented synchronous presentation at initial diagnosis [5–7] while the remainder were sequential diagnoses, often following prior treatment with alkylating agents or antimyeloma therapy [8, 9]. These observations suggest that therapy‐related myeloid neoplasia may account for a subset of cases; however, synchronous presentation in treatment‐naïve patients raises the possibility of alternative mechanisms such as age‐related clonal hematopoiesis or a shared progenitor. Herein, we report a synchronous, treatment‐naïve case of CMML and symptomatic MM to further expand the limited literature on this rare dual hematologic malignancy.

## 2. Case Presentation

A 42‐year‐old male with no significant past medical history was referred to our hospital with progressive fatigue and generalized weakness for one month, accompanied by constitutional symptoms. He had no previous exposure to cytotoxic chemotherapy, radiotherapy, or immunomodulatory therapy. There was no history of autoimmune disease or prior monoclonal gammopathy.

**FIGURE 1:**
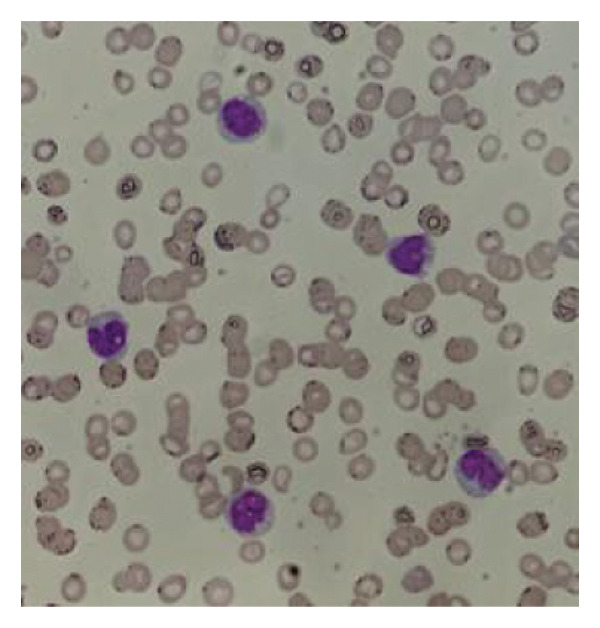
Peripheral blood smear showing marked absolute monocytosis with atypical monocytes exhibiting nuclear irregularity and cytoplasmic vacuolation (Wright–Giemsa stain, × 400).

Initial laboratory investigations revealed leukocytosis with a total white blood cell count of 22.08 × 10^9^/L, hemoglobin of 9.1 g/dL, and a platelet count of 263 × 10^9^/L. The differential count showed marked monocytosis with 74.2% of leukocytes, which is equivalent to an absolute monocyte count of 16.4 × 10^9^/L. Absolute neutropenia with an absolute neutrophil count of 0.20 × 10^9^/L was also evident (Table 1). Significant monocytosis with dysplastic monocytes and circulating nucleated red blood cells was confirmed by peripheral blood smear examination (Figure 1).

**TABLE 1:** Initial laboratory and diagnostic findings at presentation.

Parameter	Result	Reference range
White blood cell count	22.08 × 10^9^/L	4.0–11.0 × 10^9^/L
Hemoglobin	9.1 g/dL	13.0–17.0 g/dL
Platelet count	263 × 10^9^/L	150–400 × 10^9^/L
Absolute monocyte count	16.4 × 10^9^/L	0.2–0.8 × 10^9^/L
Monocyte percentage	74.2%	2%–10%
Absolute neutrophil count	0.20 × 10^9^/L	1.5–7.5 × 10^9^/L
Peripheral blood smear	Dysplastic monocytes and circulating nucleated red blood cells	—
Bone marrow cellularity	Approximately 90%	Age‐dependent
Bone marrow blasts	10%–15%	< 5%
Bone marrow plasma cells	10%–15%	< 5%
Flow cytometry	Expanded abnormal monocytic population (∼57% of total events)	—
Serum protein electrophoresis	Monoclonal spike in gamma region	Absent
M‐protein concentration	3.5 g/dL	Absent
Gamma fraction	34.2%	11%–22%
Total serum protein	10.35 g/dL	6.4–8.3 g/dL
Albumin/globulin ratio	0.59	1.0–2.1
Serum immunofixation	Monoclonal IgG kappa paraprotein	Negative
Serum free light chain ratio (*κ*/*λ*)	2.6	0.26–1.65
Imaging findings	Left lower lobe mass and lytic lesion in left femoral head	—
Lung mass biopsy	Extramedullary plasmacytoma with kappa restriction	—
Cytogenetics	45,XY,‐7 (monosomy 7)	Normal male karyotype: 46,XY

**FIGURE 2:**
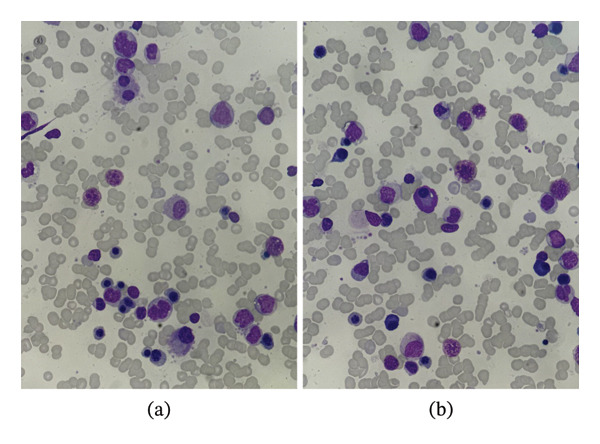
Bone marrow aspirate smears showing prominent monocytic proliferation with dysplastic features, alongside conspicuously increased plasma cells, some exhibiting binucleation and mild cytologic atypia (Wright–Giemsa stain, × 400).

Bone marrow aspirate smears were hypercellular for the patient’s age with myeloid predominance and a significant increase in monocytic lineage. Dysplastic features were noted in the megakaryocytic lineage, characterized by small hypolobated forms and multiple separate nuclei. Blasts constituted 10%–15%. In addition, a prominent increase in plasma cells was observed. The plasma cells exhibited occasional binucleation and mild cytologic atypia (Figure 2).

**FIGURE 3:**
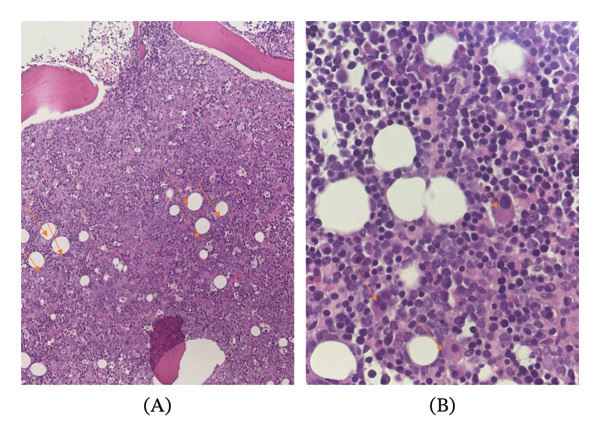
Bone marrow core biopsy. (A) Markedly hypercellular marrow (∼90% cellularity) with diffuse reduction of marrow fat spaces (orange arrows) and expansion of the myelomonocytic lineage (H&E, × 200). (B) Prominent dysmegakaryopoiesis with hypolobated and morphologically atypical megakaryocytes (orange arrows) (H&E, × 400).

**FIGURE 4:**
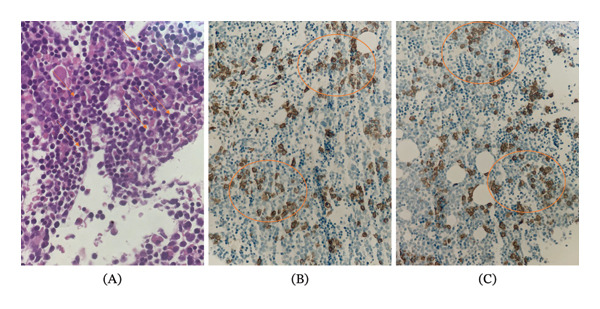
Plasma cell expansion in the bone marrow. (A) H&E‐stained core biopsy section showing increased interstitial plasma cells (orange arrows) (x 200). (B and C) CD138 immunohistochemistry highlighting increased plasma cells forming focal aggregates (orange circles) and interstitial infiltrates, supporting a plasma cell neoplasm in the appropriate clinical context (× 200).

Bone marrow core biopsy confirmed a markedly hypercellular marrow (approximately 90% cellularity) with prominent monocytic proliferation and dysmegakaryopoiesis (Figure 3A,B). Immunohistochemical staining for CD61 highlights micromegakaryocytes. CD34 immunostaining demonstrated 10%–15% blasts. CD138 immunostaining highlighted increased plasma cells (10%–15%) in an interstitial distribution with focal aggregates (Figure 4A–C).

**FIGURE 5:**
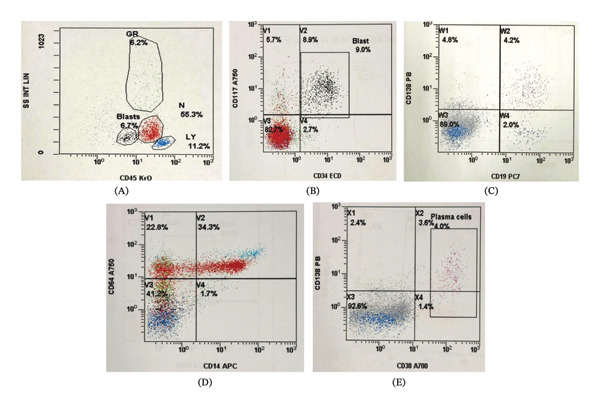
Multiparametric flow cytometric immunophenotyping of bone marrow aspirate. (A) CD45 versus side scatter plot showing expansion of the monocyte population. (B and C) Abnormal monocytic population expressing CD14 and CD64 with aberrant absence of CD16. (D) Distinct plasma cell population exhibiting bright CD38 and CD138 expression. (E) Plasma cells demonstrating expression of CD19.

Multiparametric flow cytometric immunophenotyping of the bone marrow aspirate revealed approximately 9% blasts expressing CD34, CD117, CD13, CD33, CD58, HLA‐DR, and dim CD4. A markedly expanded abnormal monocytic population accounting for approximately 57% of total events was identified. These cells expressed CD14, CD64, CD33, CD13, CD11b, CD11c, CD38, CD58, CD4, and HLA‐DR, with dim CD15 expression and absence of CD16. The expression of cytoplasmic myeloperoxidase was dim. The immunophenotypic profile supported a clonal abnormal monocytic proliferation consistent with CMML (Figure 5A–C). In addition, a plasma cell population was identified (4% of marrow cells), characterized by bright CD38 and CD138 expression. These cells were positive for CD19 and showed aberrant expression of CD56. Light‐chain restriction was not assessed by flow cytometry (Figure 5D,E). Collectively, the morphologic, immunophenotypic, and cytogenetic findings supported the presence of a clonal myeloid neoplasm consistent with CMML coexisting with a plasma cell neoplasm.

**FIGURE 6:**
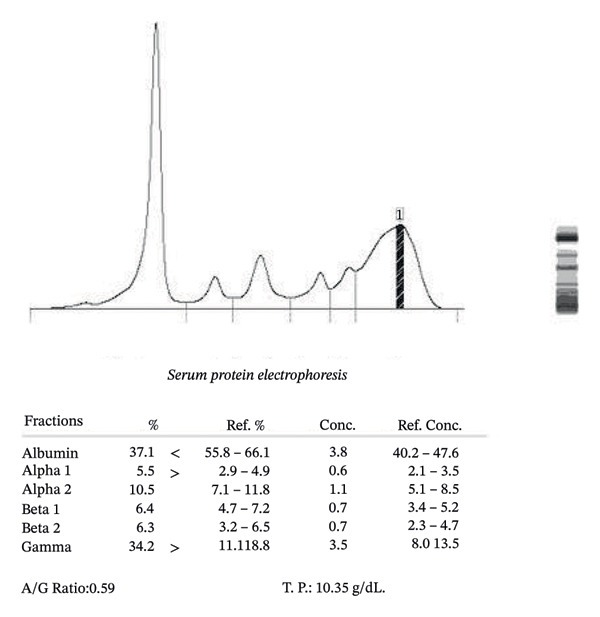
Serum protein electrophoresis demonstrates a sharp monoclonal spike in the gamma region (3.5 g/dL). The gamma fraction comprises 34.2% of total serum proteins, with elevated total protein (10.35 g/dL) and a decreased albumin‐to‐globulin ratio (A/G ratio 0.59), consistent with a monoclonal gammopathy.

In view of the increased number of plasma cells in the bone marrow biopsy, serum protein electrophoresis was performed, which revealed a significant monoclonal spike in the gamma region. The gamma fraction represented 34.2% of the total serum proteins and equated to a concentration of 3.5 g/dL. Total serum protein was high at 10.35 g/dL, accompanied by a low albumin‐to‐globulin ratio at 0.59 (Figure 6). These findings were consistent with a significant monoclonal gammopathy. Serum immunofixation revealed a monoclonal IgG kappa paraprotein. Serum FLC assay showed a total kappa/total lambda ratio of 2.6.

Imaging studies revealed a 5.0 × 2.8 × 2.8 cm spiculated mass in the superior segment of the left lower lung lobe, accompanied by a left‐sided pleural effusion. CT‐guided biopsy of the lesion showed a plasma cell‐rich infiltrate positive for CD138 with kappa light‐chain restriction on immunohistochemistry, suggestive of extramedullary plasmacytoma in the right clinical context. There was also a small (8 mm) lytic bone lesion with peripheral sclerosis in the left femoral head.

**FIGURE 7:**
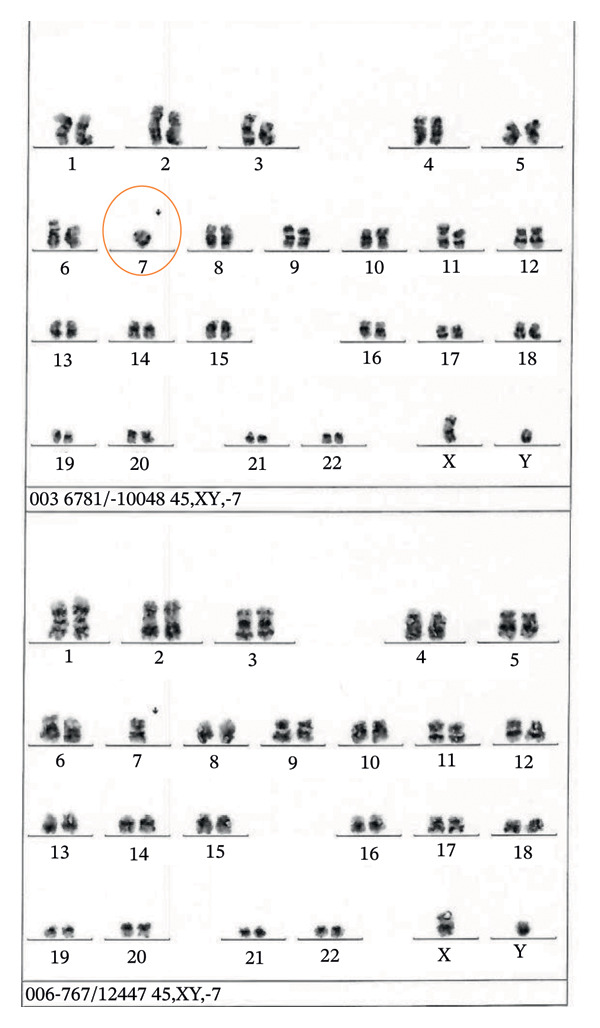
Conventional cytogenetic analysis demonstrated monosomy 7 (orange circle) with a karyotype of 45,XY,‐7, supporting the presence of a clonal myeloid neoplasm.

Conventional cytogenetic analysis identified monosomy 7 with a karyotype of 45,XY,‐7, indicating a clonal myeloid neoplasm (Figure 7).

**FIGURE 8:**
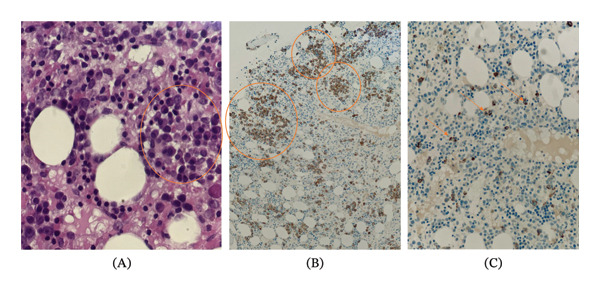
Follow‐up bone marrow evaluation after one cycle of azacitidine. (A) H&E‐stained bone marrow core biopsy showing a prominent increase in plasma cells (orange circle), accounting for approximately 20%–25% of marrow cellularity (× 200). (B) CD138 immunohistochemistry highlighting the expanded plasma cell population with increased aggregates (orange circles) (× 200). (C) CD34 immunostaining demonstrating a reduced blast population (∼7%) (orange arrows) compared with the initial evaluation (× 200).

In view of the absolute monocytosis, dysmegakaryopoiesis, an expanded abnormal monocytic population by flow cytometry, and the presence of monosomy 7, a diagnosis of CMML‐2 was made. Concurrently, the presence of bone marrow plasmacytosis (10%–15%) with a significant IgG kappa monoclonal spike, associated with a lytic bone lesion and biopsy‐proven extramedullary plasmacytoma, supported the diagnosis of MM. These findings were consistent with synchronous presentation of CMML and plasma cell myeloma in a treatment‐naïve patient. At the time of presentation, the patient did not receive myeloma‐specific therapy, as the treating team considered CMML to be the predominant driver of the clinical manifestations and cytopenias. The patient subsequently received a cycle of azacitidine, after which cytopenias worsened. A follow‐up bone marrow examination showed a reduction in blasts to approximately 7% by CD34 immunostaining. However, plasma cells increased to approximately 20%–25% by CD138 immunostaining. Peripheral monocytosis was no longer evident (Figure 8).

## 3. Discussion

The synchronous presentation of CMML and MM in a treatment‐naïve patient is exceedingly rare. CMML is a clonal myelodysplastic/myeloproliferative neoplasm distinguished by persistent monocytosis, dysplasia, and recurrent cytogenetic and molecular abnormalities [1, 2]. In contrast, MM is a terminal B‐cell malignancy defined by clonal plasma cell proliferation and monoclonal protein production [3, 4]. The simultaneous occurrence of these two biologically distinct hematologic malignancies at initial diagnosis presents substantial pathogenetic and clinical implications.

A literature review revealed that the coexistence of CMML and MM has been documented in around 10 cases [5–9]. The majority of the reported cases occurred in elderly patients, consistent with the typical median age of CMML (73–75 years) and MM (66–70 years) [1, 3]. Furthermore, several cases emerged after exposure to alkylating agents or other cytotoxic treatment, indicating sequential diagnosis and suggesting therapy‐related myeloid neoplasia [8, 9]. Synchronous presentation at initial diagnosis in a treatment‐naïve patient, as demonstrated in our case, is notably rare. The patient’s relatively young age (42 years) emphasizes the unusual nature of this presentation and raises the possibility of underlying genetic susceptibility or unidentified predisposing molecular events. Additional cases describing synchronous CMML and plasma cell neoplasms support the concept that these dual hematologic malignancies may arise through heterogeneous pathogenetic mechanisms rather than a single uniform biologic process in the development of such dual hematologic malignancies [10, 11].

The coexistence of two separate hematologic malignancies may also be indicative of alterations within the bone marrow microenvironment and the presence of clonal hematopoiesis. Chronic inflammatory signaling, cytokine dysregulation, and genomic instability within hematopoietic stem cells may provide conditions that favor the emergence of multiple independent or divergent clonal populations [12–14]. In plasma cell myeloma, cytokines such as interleukin‐6 contribute to plasma cell proliferation and survival, whereas CMML is characterized by complex molecular and epigenetic abnormalities involving hematopoietic stem and progenitor cells [2, 14].

The coexistence of CMML and MM has been previously investigated, and multiple pathogenetic mechanisms were discussed. One possibility is the independent emergence of two distinct clonal hematologic malignancies. Given that hematopoietic progenitors at different stages of differentiation are the source of these two diseases, independent clonal evolution remains plausible. Alternatively, a shared progenitor hypothesis suggests that an early hematopoietic stem cell undergoes genetic mutations that subsequently diverge into myeloid and plasma cell lineages. The presence of monosomy 7 in our case supports the existence of a clonal myeloid neoplasm, as monosomy 7 is a well‐known cytogenetic abnormality in CMML that is associated with poor prognostic outcomes [1, 2]. On the other hand, monosomy 7 is not a recognized cytogenetic abnormality in plasma cell myeloma, suggesting that the plasma cell clone represents a separate malignant clone rather than a direct descendant of the CMML clone. Notably, the plasma cell population in our study demonstrated CD19 expression and aberrant CD56 expression. Light‐chain restriction was not assessed by flow cytometry, and the diagnosis of myeloma was made based on bone marrow plasmacytosis, monoclonal paraprotein, and biopsy‐proven kappa‐restricted extramedullary plasmacytoma.

Genomic instability and clonal hematopoiesis may predispose to the emergence of various hematologic malignancies and their coexistence with other hematologic diseases. However, the patient’s relatively young age makes age‐related clonal hematopoiesis less likely and instead reinforces the theory of either an early shared progenitor mutation or coincidental dual clonal events.

From a diagnostic perspective, reactive monocytosis and reactive plasmacytosis must be excluded. In our case, the presence of persistent absolute and relative monocytosis, dysplastic marrow megakaryopoiesis, an expanded abnormal monocytic population identified by flow cytometry, and the cytogenetic evidence of monosomy 7 supported a clonal myeloid neoplasm consistent with CMML. The identification of 10%–15% plasma cells in the bone marrow biopsy, a significant IgG kappa monoclonal spike (3.5 g/dL) on protein electrophoresis, and an associated lytic bone lesion and biopsy‐proven extramedullary plasmacytoma confirmed the diagnosis of symptomatic MM rather than reactive plasmacytosis or monoclonal gammopathy of undetermined significance.

The simultaneous presence of CMML and MM complicates treatment. In fact, hypomethylating agents used to treat CMML may not adequately control the myeloma component of the disease, and conventional antimyeloma treatment regimens may exacerbate cytopenias or accelerate myeloid disease progression. Finally, the presence of monosomy 7 is a poor prognostic indicator in the context of CMML and may possibly influence treatment decisions and transplant considerations. Conventional cytogenetic analysis in our case showed monosomy 7, supporting the presence of a clonal myeloid process. However, comprehensive molecular testing for CMML‐associated mutations was unavailable at our institution; therefore, additional molecular risk stratification could not be performed. Myeloma‐specific FISH studies were not assessed; therefore, abnormalities such as t(11; 14)/CCND1 rearrangement could not be evaluated. Identification of such abnormalities may have therapeutic implications, including potential consideration of venetoclax‐based therapy. Therefore, tailored and multidisciplinary treatment approaches are crucial in these cases. The patient received a single cycle of azacitidine treatment with subsequent worsening cytopenias. This demonstrates the therapeutic challenges in addressing dual myeloid and plasma cell malignancies. The deterioration in cytopenias after hypomethylating therapy may be attributed to azacitidine’s limited ability to control the plasma cell component and its adverse effect on the residual hematopoiesis. These dynamics emphasize the lack of standardized treatment protocols for coexisting CMML and MM, underscoring the necessity of individualized, multidisciplinary therapeutic planning.

## 4. Conclusion

Given the rarity of synchronous CMML and MM, systematic reporting and molecular characterization of additional cases will be essential to clarify the potential pathogenetic connections and optimal therapeutic approaches. In summary, we report an unusual case of concurrent CMML and MM in a young, untreated patient with monosomy 7. This case expands the limited literature on concurrent hematologic malignancies and underscores the necessity of comprehensive marrow evaluation when unusual or inconsistent diagnoses arise. The recognition of simultaneous clonal diseases has considerable diagnostic, treatment, and outcome implications.

**Nomenclature**
CMML: Chronic myelomonocytic leukemia
MM: Multiple myeloma
CRAB: Hypercalcemia, renal insufficiency, anemia, bone lesions
FLC: Free light chain
WHO: World health organization
IARC: International agency for research on cancer
BM: Bone marrow
WBC: White blood cell
Hb: Hemoglobin
MCV: Mean corpuscular volume
RDW: Red cell distribution width
SSC: Side scatter
H&E: Hematoxylin and eosin

## Author Contributions

Moutaz W. Sweileh contributed to case diagnosis, data collection, and manuscript drafting. Naim Qamhia contributed to case diagnosis and critically revised the manuscript. Amal Batta performed and interpreted the flow cytometry analysis. Hisham Asaad, Razan Yousef Odeh, and Mamoun Swaileh contributed to the clinical management of the patient.

## Funding

This study did not receive any type of financial funding throughout all stages of the project.

## Disclosure

All authors read and approved the final manuscript.

## Ethics Statement

This study was reviewed and approved by the Institutional Review Board of An‐Najah National University Hospital.

## Consent

Informed consent for publication of this case report and accompanying images was obtained from the patient. The Institutional Review Board approved the use of verbal consent in accordance with institutional policies.

## Conflicts of Interest

The authors declare no conflicts of interest.

## Data Availability

The data supporting the findings of this case report are available from the corresponding author upon reasonable request, in accordance with institutional policies and patient confidentiality regulations.

## References

[1] Board W. C. T. E. , WHO Classification of Tumours Editorial Board: Chronic Myelomonocytic Leukemia, WHO Classification of Tumours: Haematolymphoid Tumours, 2022, 5th edition, International Agency for Research on Cancer (IARC), Lyon, France.

[2] Patnaik M. M. and Tefferi A. , Chronic Myelomonocytic Leukemia: 2024 Update on Diagnosis, Risk Stratification and Management, American Journal of Hematology. (2024) 99, no. 6, 1142–1165, 10.1002/ajh.27271. 38450850 PMC11096042

[3] Kazandjian D. , Multiple Myeloma Epidemiology and Survival: A Unique Malignancy, Seminars in Oncology. (2016) 43, no. 6, 676–681, 10.1053/j.seminoncol.2016.11.004. 28061985 PMC5283695

[4] Board W. C. T. E. , WHO Classification of Tumours Editorial Board: Plasma Cell Myeloma/Multiple Myeloma, WHO Classification of Tumours: Haematolymphoid Tumours 2022, 2022, 5th edition, International Agency for Research on Cancer, Lyon, France.

[5] Habashi H. M. , Sharp R. A. , and Pippard M. J. , Multiple Myeloma and Chronic Myelomonocytic Leukaemia Developing in a Patient With Autoimmune Disease, Journal of Internal Medicine. (1991) 230, no. 4, 361–362, 10.1111/j.1365-2796.1991.tb00457.x. 1919430

[6] Raz I. and Polliack A. , Coexistence of Myelomonocytic Leukemia and Monoclonal Gammopathy or Myeloma. Simultaneous Presentation in Three Patients, Cancer. (1984) 53, no. 1, 83–85, 10.1002/1097-0142(19840101)53:1<83::aid-cncr2820530115>3.0.co;2-q. 6360331

[7] Siddiqui W. T. , Farhan M. , and Nguyen K. , To Treat or Not to Treat: A Case of Simultaneous Discovery of Chronic Myelomonocytic Leukemia and Multiple Myeloma, Cureus. (2021) 13, no. 8, 10.7759/cureus.17505. PMC8476195 34603882

[8] Natazuka T. , Yamaguchi T. , Murayama T. et al., Chronic Myelomonocytic Leukemia Following Prolonged Alkylating Agent Therapy for Multiple Myeloma, International Journal of Hematology. (1994) 60, no. 4, 263–265. 7894029

[9] Ueki K. , Sato S. , Tamura J. et al., Three Cases of Multiple Myeloma Developing Into Melphalan-Related Chronic Myelomonocytic Leukemia, Journal of Medicine. (1991) 22, no. 3, 157–161. 1770323

[10] Arber D. A. , Orazi A. , Hasserjian R. P. et al., International Consensus Classification of Myeloid Neoplasms and Acute Leukemias: Integrating Morphologic, Clinical, and Genomic Data, Blood. (2022) 140, no. 11, 1200–1228, 10.1182/blood.2022015850. 35767897 PMC9479031

[11] Patnaik M. M. , How I Diagnose and Treat Chronic Myelomonocytic Leukemia, Haematologica. (2022) 107, no. 7, 1503–1517, 10.3324/haematol.2021.279500. 35236051 PMC9244829

[12] Caiado F. and Manz M. G. , Clonal Hematopoiesis: Impact on Health and Disease, Hematological Oncology. (2025) 43, no. Suppl 2, 10.1002/hon.70075. PMC12167641 40517440

[13] Sperling A. S. , Gibson C. J. , and Ebert B. L. , The Genetics of Myelodysplastic Syndrome: From Clonal Haematopoiesis to Secondary Leukaemia, Nature Reviews Cancer. (2017) 17, no. 1, 5–19, 10.1038/nrc.2016.112. 27834397 PMC5470392

[14] Steensma D. P. , Bejar R. , Jaiswal S. et al., Clonal Hematopoiesis of Indeterminate Potential and Its Distinction From Myelodysplastic Syndromes, Blood. (2015) 126, no. 1, 9–16, 10.1182/blood-2015-03-631747. 25931582 PMC4624443

